# Immunogenicity and protective efficacy of an inactivated infectious bronchitis virus vaccine candidate from a local isolate of Bangladesh

**DOI:** 10.5455/javar.2024.k809

**Published:** 2024-09-29

**Authors:** Mst. Kohinoor Parvin, Md. Enamul Haque, Mohammad Aynul Haque, Md. Mostofa Kamal, Mohammad Sadekuzzaman, Sajedul Hayat, Md. Tanvir Rahman, Mahbubul Pratik Siddique, Sham Soun Nahar, A. K. M. Khasruzzaman, Muhammad Tofazzal Hossain, Md. Alimul Islam

**Affiliations:** 1 Department of Microbiology and Hygiene, Bangladesh Agricultural University, Mymensingh, Bangladesh; 2 Central Disease Investigation Laboratory, Department of Livestock Services, Dhaka, Bangladesh; 3 Livestock Research Institute, Dhaka, Bangladesh

**Keywords:** Infectious bronchitis, inactivated IB vaccine, live IB vaccine, immunogenicity, antibody titers, protective efficacy

## Abstract

**Objective:**

Infectious bronchitis (IB), a highly infectious acute viral disease, is a major burden to the chicken industry worldwide. The research aimed to develop an inactivated IB vaccine using local isolates and assess its immunogenicity compared to other commercial live IB vaccines.

**Materials and Methods:**

An inactivated vaccine using a candidate IB virus (PP067159.1: Alim_IB_1001) of the QX genotype was developed according to WOAH guidelines. Chickens were vaccinated with three doses (0.25, 0.5, and 1.0 ml) at 7 days old, with a booster at 37 days old via subcutaneous (SC) and intramuscular (IM) routes. Blood samples were collected on days 7, 37, and 67 to measure immune response by indirect ELISA. On day 67, chickens were challenged with a virulent IBV strain to assess vaccine protection. The experimental IB vaccine’s immunogenicity, protective efficacy, and antibody duration were compared to a live IB vaccine (Live CEVAC^®^ IBird) using three vaccination schedules: killed-followed-killed, live-followed-killed, and live-followed-live.

**Results:**

Chickens vaccinated with SC with 1.0 ml showed higher antibody titers compared to other SC and IM routes of vaccination. SC vaccination with 0.5 and 1 ml provided the highest protection (93%). The killed-followed-killed vaccination method produced a more consistent and protective level of antibody titers in chickens compared to the other vaccination schedules. The experimental inactivated IB vaccine led to a higher survival rate (93%) compared to live-followed-killed (87%) and live-followed-live (73%), with statistical significance (*p < 0.01*). All three chicken groups maintained protective antibody titers (>396) at 307 days, but titers declined faster in the live-followed-live and live-followed-killed groups compared to the killed-followed-killed group.

**Conclusion:**

The study found that the experimental inactivated IB vaccination can protect commercial-layer chickens from natural IB outbreaks of the QX genotype.

## Introduction

Infectious bronchitis (IB) is a highly transmitted and economically significant avian disease that affects the poultry industry globally [1]. IB virus (IBV), a member of the *Coronaviridae* family, is responsible for this disease. It primarily affects the respiratory system, causing breathing difficulties, damaging the kidneys, and reducing egg production and quality [2]. The morbidity rate is 100% in flocks that have not received vaccinations. The mortality rate is dependent on the specific strain of the virus, with a maximum of 60% observed in flocks that have not been vaccinated [3]. This significantly negatively impacts the poultry industry’s profitability and long-term viability [4]. To effectively control IB in poultry, a thorough strategy is necessary, which includes implementing proper management practices, enforcing strict biosecurity measures, administering vaccinations, and occasionally providing treatment [3]. Although strict biosecurity measures are in place, IBV remains a widespread threat because of its rapid mutation rate. This results in the development of new serotypes and variants that can avoid the immune responses triggered by current vaccinations [5].

Developing effective vaccines against IBV is complicated by the virus’s genetic variability and the limited cross-protection provided by various strains [6]. Conventional vaccines, mainly consisting of live attenuated and killed (inactivated) variants, have been the mainstay of IBV control [7]. Nevertheless, due to the dynamic characteristics of the virus, it is necessary to consistently evaluate and create novel vaccines that can offer more comprehensive immunity against various serotypes, particularly local variants that may not be adequately targeted by commercially available vaccinations designed for global strains. The four structural proteins of the virus—spike (S), membrane (M), small envelope (E), and nucleocapsid (N)—are the primary targets for vaccine development. The spike protein (S) is the most immunogenic for IB vaccines. Recent advances include “RG” chimeric IBV strains and virus-like particles which show promise for broad protection [8, 9]. Inactivated vaccines use affinity-purified IBV antigens or chemically inactivated viruses, typically of the Mass-type genotype. Multivalent inactivated vaccines and live vaccines have effectively enhanced protection against multiple IBV strains. Research has also demonstrated the efficacy of several substances, such as chitosan nanoparticles and resiquimod, in boosting the immune response to vaccines [8, 10]. Live vaccines are generally more effective than inactivated ones, with studies showing cross-protection against different virus challenges in animals vaccinated with attenuated mass vaccines [5, 11].

This study aims to investigate the immune response produced by recently developed inactivated vaccines made from local IBV isolates. The experimental vaccines are developed to enhance immune responses against common diseases in specific geographic areas. Prioritizing local isolates due to their genetic and antigenic similarity to current poultry viruses could lead to more efficient protection against this virus. The study also determined the comparative efficacy, protection study, and duration of immune response to in-house inactivated IB and commercial live IB vaccines.

## Materials and Methods

### Ethical statement

The experiment ensured conformity with established ethical standards by closely adhering to the parameters given by Bangladesh Agricultural University in Mymensingh’s Animal Welfare and Experimentation Ethics Committee (AWEEC). It received formal authorization, indicated by the reference number [Ref. No. AWEEC/BAU/2021(52)].

### Selection of chickens for experimental study

Brown-layer healthy day-old chicks were collected from the control shed of the city hatchery in Narsingdi district, Bangladesh, regardless of their levels of maternal antibodies (S/P≤0.2 or serum antibody titer ≤396: negative). The chicks were reared in an isolated shed throughout the experiment, with sufficient water and feed provided while maintaining strict biosecurity (spraying anti-viral solution thrice a day).

### Selection of strain, propagation, and virus titration

The QX genotype of the IBV strain (PP067159.1: Alim_IB_1001) was selected as a vaccine candidate after a comprehensive assessment of its molecular, serological, and biological characteristics and pathogenicity [12]. In order to achieve large-scale manufacturing, the selected IBV strain was propagated in seronegative embryonated chicken eggs (ECEs) that were 9 days old using the allantoic cavity route. Centrifugation was done to clear the non-viral embryonic debris from the allantoic fluid harvested aseptically, which was subjected to 5,000 rpm for 10 min at 4°C. The subsequent transparent fluid was collected. The tenth passage of the candidate IBV isolate was selected for large propagation for vaccine production. To determine 50% of the embryo infectious doses (EID_50_), the Reed and Muench technique [13] was utilized.

### Sterility and purity tests of the allantoic fluid

The collected allantoic fluid was cultured in bacteriological, fungal, and mycoplasma growth media and checked daily for a week to detect any microbial growth [14]. The allantoic fluid was also checked for hemagglutination (HA) pattern using the slide HA test according to the method of the WOAH manual [14]. An Reverse transcription-polymerase chain reaction (RT-PCR) test was performed to establish the purity of the IBV virus according to the method of Parvin et al. [12].

### Virus inactivation and vaccine formulation

The virus was inactivated using a concentration of 0.1% formalin (37% formaldehyde) for 24 h at room temperature.. Randomly selected samples from each batch were injected into eggs and passed through the process at least three times to confirm the virus’s inactivation. Once fully inactivated, the viruses were emulsified in an aqueous phase of Montanide (ISA-70 mineral emulsion, SEPPIC, France) at a ratio of 30:70 (w/w), following the manufacturer’s instructions. Each dosage of the vaccine emulsion contained 0.5 ml (10^7.67^ EID_50_), similar to the virus concentration of commercial live IB vaccines.

### Sterility and safety tests of inactivated IB vaccine

The IB vaccine was cultured in aerobic and anaerobic bacterial, fungal, and mycoplasma growth media. The cultures were monitored daily for a week to check microbial growth. 21-day-old ten specific pathogen-free chickens received a double dose of the vaccine via subcutaneous (SC) injection. The chickens underwent a comprehensive examination 21 days after the injection to identify any abnormal vaccination-related reactions [14].

### Determination of efficacy and protection potentiality of vaccines depends on the dose and route of administration

In this study, a total of 140 chicks at 7 days old were selected. These chicks were evenly divided into seven groups, with each group consisting of 20 chickens. The groups were designated as follows:

- Group A received a vaccination of 0.25 ml per chicken/SC route.
- Group B was vaccinated with 0.25 ml per chicken/intramuscularly (IM) route.
- Group C received 0.5 ml per chicken/SC route of vaccination.
- Group D vaccinated with 0.5 ml per chicken/IM route of vaccination
- Group E received 1.0 ml per chicken/SC route of vaccination.
- Group F was vaccinated with 1.0 ml per chicken/IM route of vaccination.
- Group G served as the unvaccinated control group.
- The priming of chickens with primary vaccination was done at 7 days old, followed by boosting through secondary vaccination at 37 days of age, except for the control group.
- Blood samples from all groups of chickens were collected at three different time points: on day 7, on day 37, and on day 67 of the chickens’ age. The collected serum was then analyzed for antibody titer using an indirect ELISA Kit (IDEXX, USA) according to the manufacturer’s instructions.
- In addition, each group of chickens, including the control group, was exposed to a challenge with a highly virulent IBV field strain of QX genotype [12] to test the vaccine’s efficacy against infection.
- On day 67, the virulent IBV strain was administered at a dose and concentration of 0.5 ml (10^3.0^ EID_50_/ml) via the IM route in an isolated environment.
- Observations were made over the next 14 days after inoculation of viruses and monitored for the development of any local or systemic reactions and any clinical signs that could indicate an IBV infection.

### Comparative efficacy and protective potentiality of the experimentally developed inactivated IB and commercial live IB vaccines

In this study, a total of 150 chicks of 7 days old were selected. These chicks were categorized into three groups, each consisting of 50 chickens. The vaccination protocol for each group was as follows:

- Chickens in group A received double doses of vaccination with a killed IB vaccine. Each dose was administered SC with a volume of 0.5 ml. The vaccine contained 50 μg of viral protein per dose. The protein concentration was determined using the LAMDA 365 UV-VIZ Spectrophotometer (Perkin Elmer, USA), following the method described by Nazari et al. [15].
- Chickens of group B were first vaccinated with a live IB vaccine (Live CEVAC^®^ IBird, Philippines), one drop containing 10^3.5^ EID_50_ of IBV, administered intraocularly, followed by a killed IB vaccine with a similar dose as that of group A.
- Chickens of group C were vaccinated twice with the live IB vaccine, each time receiving one drop, as the live IB vaccine used for group B.
- The priming of chickens by primary vaccination at 7 days of age and boost for secondary vaccination at 37 days of age.
- Blood samples of the chickens in the experimental groups were collected before and after vaccination on day 7, day 37, and day 67. These samples were then processed to collect serum for determining antibody titer using an indirect ELISA Kit (IDEXX, USA).
- On day 67, a total of fifteen chickens from each of the three vaccinated groups were subjected to challenge with a virulent strain of local isolate of IBV (QX genotype) that was homologous to their vaccine strain. Each chicken received 0.5 ml of the virus (10^3.0 ^EID_50_/ml) via the IM route of inoculation.
- After a virulent challenge with a homologous strain of the IB virus (QX genotype), chickens of all the experimental groups were observed daily for 14 days to assess the rate of survivability and development of any specific signs and symptoms of IB.

### Determination of retention period of antibody titer of ­chickens after vaccination with the experimentally ­developed inactivated and live commercial IB vaccines

The remaining vaccinated chickens were assessed for their serum antibody titers up to 307 days of age. Blood samples were taken at days 97, 127, 187, 217, and 307 days of age. The serum samples were processed for the determination of antibody titer by an indirect ELISA Kit (IDEXX, USA).

### Statistical analysis

The data obtained from this study were used for statistical analysis using analysis of variance and a *t*-test, which paired the means of two samples. This analysis was conducted using a computerized statistical program, specifically statistical package for the social sciences.

## RESULTS

### Sterility and purity test of harvested allantoic fluid

The sterility test on the harvested allantoic fluid confirmed that bacteria, fungi, or mycoplasma did not grow in nutrient agar, SS agar, blood agar, EMB agar, MacConkey agar, Sabouraud dextrose agar, or mycoplasma agar (PPLO). Additionally, the allantoic fluid showed HA activity on a slide HA test that contained IBV, which were confirmed by RT-PCR using S1 gene-specific primers.

### Inactivation, sterility, and safety tests of the developed vaccine

The absence of embryo mortality and subsequent hemagglutinating activity in allantoic fluid following inoculation into ECEs indicated that the viruses were inactivated completely. The sterility test showed that the experimentally developed inactivated IB vaccine was free from all kinds of contamination, as confirmed by inoculating it onto the bacteriological, fungal, and mycoplasma growth media. In the safety test, no deaths, local and systemic tissue reactions, or clinical signs of IBV were found in the vaccinated chickens.

### Dose and route-dependent immune response of the newly developed inactivated IB vaccine

**Figure 1.**
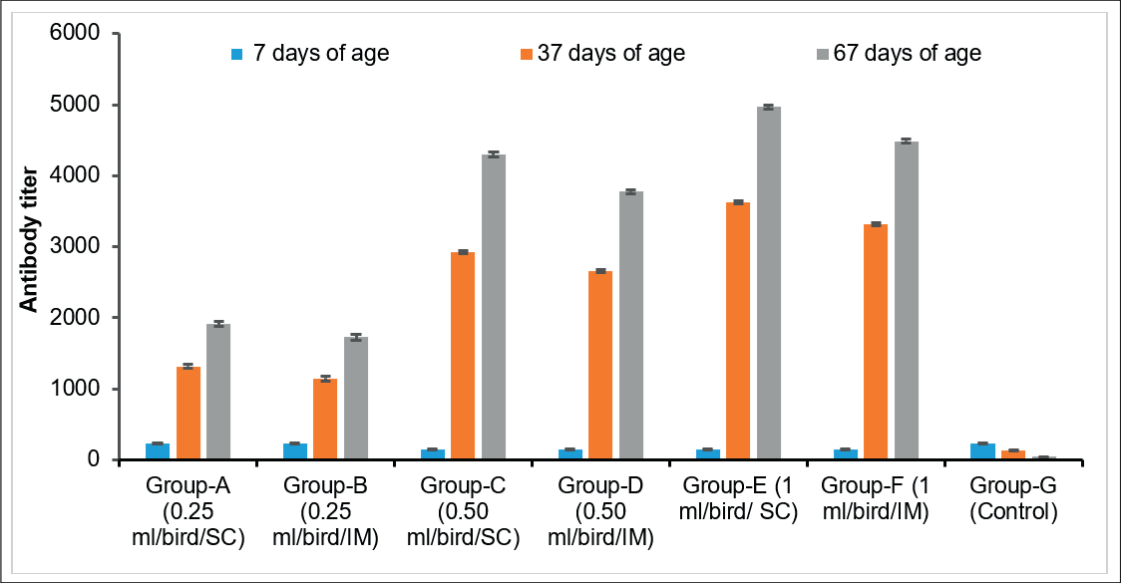
Dose and route-dependent serum antibody titer of IB vaccinated and non-vaccinated chickens.

The birds in all seven groups had antibody levels that ranged from 151.12 ± 4.14 to 233.29 ± 4.00 before they were vaccinated (Fig. 1). Still, the post-vaccination antibody titers of serum from the chickens in this study group, collected on days 37 and 67, varied significantly (*p *< 0.05). Chicken of Group E, which received 1.0 ml per dose via the SC route, showed a better immune response, reaching an ELISA titer of 3626.342 ± 25.70 to 4969.251 ± 30.87 on days 37 and 67, respectively. Chicken of Group C, which received 0.50 ml per dose via the same SC route, showed the antibody titer reaching from 2926.74 ± 25.70 to 4299.47 ± 30.86 on days 37 and 67, respectively. In contrast, chickens belonging to group A were vaccinated with a dose of 0.25 ml per chicken administered SC route, exhibited the lowest antibody response among the vaccinated groups, with titers of 1318.375 ± 31.41 on day 37 and 1913.324 ± 35.30 on day 67. Chicken of Group C, which received 0.50 ml per dose via the same SC route, showed the antibody titer 2926.74 ± 25.70 on day 37 by ELISA. Still, chickens of Group D, which received the same dose via the IM route, showed an antibody titer of 2656.74 ± 24.67 on day 37. However, on day 67, chickens of Group C showed the antibody titer 4299.47 ± 30.86 for the SC route and 3776.47 ± 26.61 for the IM route of Group D, respectively. There was no significant difference (*p* > 0.05) in serum antibody titers of chicken blood collected on days 37 and 67 immunized via the SC and IM routes in this study (Fig. 1).

**Table 1.** Dose and route-dependent protective potentiality of the experimentally developed inactivated IB vaccine.

Experimental groups	Protection rate (%)
Group-A (0.25 ml/chicken/SC)	67 ± 5.77
Group-B (0.25 ml/chicken/IM)	63 ± 5.77
Group-C (0.50 ml/chicken/SC)	93 ± 5.77
Group-D (0.50 ml/chicken/IM)	83 ± 5.77
Group-E (1 ml/chicken/ SC)	93 ± 5.77
Group-F (1 ml/chicken/IM)	87 ± 5.77
Group-G (Control)	33 ± 5.77

SC-subcutaneous; IM-intramuscular

### Dose and route-dependent protective study of the inactivated IB vaccine

The protection rates of chickens vaccinated with different doses and routes were found to be highest in Groups C and E, around 93%, and they received 0.5 ml and 1 ml per dose of vaccine/SC, respectively (Table 1). The chickens of Group B that received 0.25 ml/chicken/IM showed the lowest, around 63%, among the vaccinated group. Chickens in the control group also showed 33% protection. The protection rate among different vaccination groups was statistically significant (*p *< 0.01).

**Figure 2.**
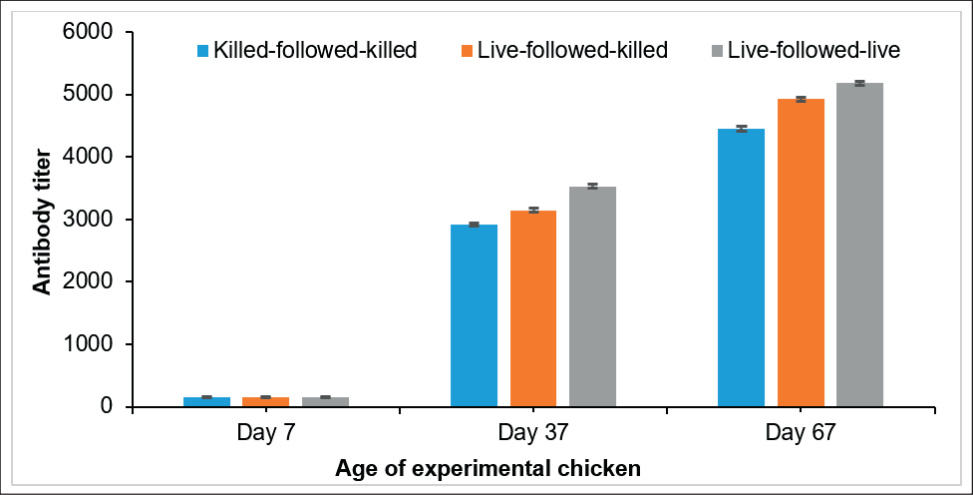
Serum antibody titer of chicken vaccinated with experimentally developed inactivated IB and commercial live IB vaccines.

**Table 2.** Protection rate of chickens after booster vaccination with experimentally developed inactivated and live commercial IB vaccines.

Experimental groups	Protection rate (%)
Killed-followed-killed	93 ± 5.77
Live-followed-killed	87 ± 5.77
Live-followed-live	73 ± 5.77

### Comparative efficacy of experimentally developed inactivated and commercial live IB vaccines

The pre-vaccination titers were 151.12 ± 4.05 for killed-followed-killed, 160.45 ± 4.13 for live-followed-killed, and 154.675 ± 4.36 for live-followed-live (Fig. 2). On day 37, the serum antibody titers of chicken were found to be 2918.37 ± 29.56 for killed-followed-killed, 3146.74 ± 29.56 for live-followed-killed, and 3526.443 ± 33.06 for live-followed-live vaccination. However, on day 67, the serum antibody titers of chickens were found to be 4453.06 ± 36.84 for killed-followed-killed, 4926.56 ± 30.17 for live-followed-killed, and 5183.369 ± 32.58 for live-followed-live immunization, respectively. On days 37 and 67, the serum antibody titers were found to be highest, around 3526.443 ± 33.06 and 5183.369 ± 32.58 in the chickens vaccinated with live-followed-live vaccination. On days 37 and 67, the chicken vaccinated with the killed-followed-killed group experienced a remarkable decrease in antibody levels of around 2918.37 ± 29.56 and 4453.06 ± 36.84, respectively (Fig. 2). There was a statistically significant variation (*p *< 0.01) in the serum antibody titer of chicken’s blood collected before vaccination (on day 7) and after vaccination (on days 37 and 67). On the contrary, no significant variation (*p *> 0.05) was noticed in the antibody titer of all three vaccinated groups of chickens.

### Protective potentiality of prepared inactivated IB with commercial live IB vaccines

The protection rates of chickens vaccinated with killed-followed-killed, live-followed-killed, and live-followed-live were found to be 93%, 87%, and 73%, respectively (Table 2). The protection rate of chickens receiving the three vaccination schedules was statistically significant (*p *< 0.01).

### Retention of the protective level of antibody titer of chickens following three vaccination schedules

**Figure 3.**
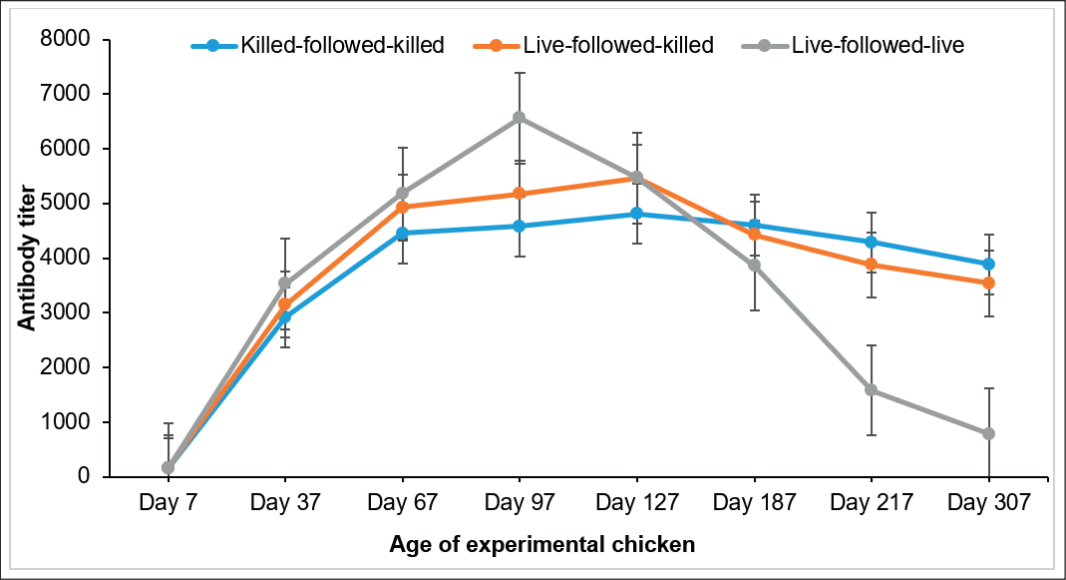
Duration of serum antibody titer of chicken vaccinated with three vaccination schedules at different time intervals.

The serum antibody titer of the chicken collected on day 97 was determined as 4,578.92 ± 40.56 for the killed-followed-killed group, 5,178.348 ± 35 for the live-followed-killed group, and 5,466.36 ± 32.94 for the live-followed-live group, respectively (Fig. 3). On day 127, the serum antibody titers of chickens in all the groups following the three schedules of vaccination were approximately 4,807.19 ± 30.99 for killed-followed-killed, 5,466.02 ± 32.17 for live-followed-killed, and 6,562.34 ± 33.12 for live-followed-live. The antibody titers of chickens in all groups started gradually declining after 127 days of age. The serum antibody titers were found to be 4,429.53 ± 34.82 for live-followed-killed, 4,603.53 ± 35.31 for killed-followed-killed, and 3,862.09 ± 32.65 for live-followed-live on day 187. All three vaccinated groups of chickens retained the protective level (>396) of serum antibody titer for up to 307 days of age. On day 307, the serum antibody titers of chickens were approximately 3,887.07 ± 35.42 for killed-followed-killed, 3,537.01 ± 36.56 for live-followed-killed, and 786.976 ± 30.57 for live-followed-live vaccination schedules (Fig. 3). The study revealed a significant variation (*p *< 0.05) in antibody titer between chicken groups vaccinated with the killed-followed-killed and live-followed-live schedules. However, this study found no significant difference (*p* > 0.05) in antibody titers between the chicken groups vaccinated using the killed-followed-killed and live-followed-killed schedules.

## Discussion

The IB is a contagious viral disease affecting chickens of all age groups. Controlling IB in poultry is very difficult due to the continuous mutation of the viruses and the prevalence of several genotypes and serotypes of IBV globally. Vaccination is a vital and well-established method for controlling IB in the poultry-raising countries of the world. Despite regular vaccination with live IB vaccines, the disease appears endemically among commercial poultry in Bangladesh. The development of a vaccine (either live or killed) using a locally circulating strain of IBV has not been carried out in Bangladesh yet. To combat these situations, the development of an effective inactivated vaccine with the circulating strains of IBV (matching viral antigens between the field and vaccine viruses) is the demand of the time. For this reason, the study was aimed at developing an inactivated IB vaccine locally circulating isolates (Genotype QX) and comparing its immune response and protective potentiality with live commercial IB vaccines.

The study evaluated the immune response of chickens vaccinated with three doses and two routes of inactivated IB vaccine. On days 37 and 67, chickens vaccinated through the SC route with doses of 0.25 ml/chicken, 0.5 ml/chicken, and 1.0 ml/chicken exhibited slightly higher serum antibody titers than chickens vaccinated through the IM route with the same doses. These findings are consistent with a study conducted by Woziri et al. [16], who developed an inactivated avian influenza H5 vaccine and evaluated the antibody response in chickens vaccinated with three different doses (0.2, 0.5, and 0.7 ml) using two routes (IM and SC) of administration. In their study, they observed higher antibody levels in the chicken groups vaccinated through the SC route compared to the IM route 21 days following vaccination. In this study, the protective efficacy of the prepared IB vaccine from local isolates ranged from 63% to 93% protection, depending on doses and routes after a challenge study with a homologous strain of the IBV. Fathy et al. [17] evaluated the efficacy of the bivalent IBV vaccine produced from local isolates and showed 92% to 96% protection after being challenged with a homologous virus.

Based on the immune response and protective efficacy observed with three different doses (1.0, 0.5, and 0.25 ml/chicken) and two different routes (SC and IM), the 0.5 ml/dose/chicken/SC combination was chosen for mass immunization. These doses and routes were also chosen based on the initial screening for viral antigen concentration (10^7.67^EID_50_/dose) and the cost-effectiveness of each vaccine dose. In this study, the standard dose was used for vaccination in three scheduled groups of layer chickens (killed-followed-killed, live-followed-killed, and live-followed-live).

Chickens that received two vaccinations with the live-followed-live IB vaccine had higher serum antibody titers than those immunized twice with the live-followed-killed and killed-followed-killed vaccinations. In their study, Buharideen et al. [18] assessed the effectiveness of two distinct vaccination strategies in inducing a thorough immunological response in laying chickens. The initial method employed live-attenuated IB vaccines, but the subsequent method included both live-attenuated and inactivated IB vaccines. The serum’s antibody levels against IBV were evaluated at two specific time points: three weeks and ten weeks following the last immunization. The vaccination technique, including live attenuated and inactivated vaccines, demonstrated superior efficacy in inducing both systemic and localized immune responses in the vaccinated chickens. Inactivated vaccines are commonly used in conjunction with live vaccines in most cases of commercial poultry immunization. This combination can amplify the immune response initially triggered by a live vaccine, providing enhanced and more comprehensive immunity [19]. The present study demonstrated that the locally circulating genotype QX of IBV, when used for the development of the inactivated vaccine, resulted in better immune responses and protective potentiality in the vaccinated flock of chickens. The vaccinated birds had a high percentage of protection and increased antibody titers when using double doses of inactivated vaccines made with local isolates. This approach reduces economic losses for farmers by decreasing morbidity, mortality, and productivity issues in commercial layer poultry due to IB infection [17].

The results of the challenge study showed that chickens vaccinated twice with the killed-followed-killed vaccine had a higher protection rate (93%) than chickens in the live-followed-live (73%) and live-followed-killed (87%) groups. This result is similar to Yan et al. [20], who reported that only 10% of hens died after receiving an inactivated IBVSX16 vaccine. However, the birds vaccinated with the inactivated IB vaccine provided more than 80% protection against infection by the challenge virus. Erfanmanesh et al. [21] developed a vaccine against the IBV variant 2 (IS-1494/GI-23) genotype and administered 0.5 ml per dose per chicken. During evaluation, it was found that chickens vaccinated with this variant 2-derived vaccine exhibited a 67% protection rate, slightly higher than the 60% protection rate observed with standard commercial live IB vaccines. Although the difference in protection rates was not statistically significant, the variant 2 vaccine reduced viral presence in feces and kidneys compared to the commercial live vaccine.

After booster vaccinations (on day 37) with killed-followed-killed, birds’ serum antibody titers increased and continued to increase until day 127. The antibody titer of chicken was found to be a protective level until 307 days of age. However, the serum antibody titer of chickens vaccinated twice with live-followed-killed and live-followed-live increased simultaneously and remained up to 307 days of age. All three groups of chickens vaccinated with killed-followed-killed, live-followed-live, or both exhibited serum antibody titers >396 for up to 307 days. The antibody titer declined faster in the chickens of the live-followed-live vaccination groups compared to the killed-followed-killed groups. This study compared the immunogenicity of the experimentally developed inactivated IB vaccine to the live IB vaccine using serum antibody titers by ELISA. Unlike live-followed-killed and live-followed-live, chickens vaccinated with killed-followed-killed had a higher titer than the protective level. The duration of immunity and retention period of the serum antibody titer of the chicken group vaccinated with experimentally developed inactivated IB vaccine remained above the protective level (>396) until day 307 after receiving two doses of vaccinations. The finding of the retention period of serum antibody titer of chicken vaccinated with inactivated IB vaccine aligns with the findings of Bhuiyan et al. [3], who noted that inactivated vaccinations generally produce a long-lasting and persistent immune response. This is crucial for effectively managing IB in poultry populations over an extended period.

Inactivated vaccines contain killed viruses, which cannot replicate in vaccinated birds’ bodies. The reduced risk of vaccine-induced disease is especially important for immunocompromised birds, as live vaccines can be a concern. Moreover, the utilization of inactivated viruses in the vaccine prevents the transmission of the virus from immunized to non-immunized birds, hence enhancing the safety of densely populated chicken flocks [22].

## Conclusion

The findings of this study concluded that the experimentally developed inactivated IB vaccine with the locally circulating isolate of IBV is safe, has no chance of reversion into a virulent form, and might have the ability to successfully control frequent outbreaks of IB in commercial layer poultry populations in Bangladesh. Based on the findings of this study, it can also be concluded that using double doses of an inactivated IB vaccine developed with the locally circulating strain of the genotype QX of IBV is more effective in terms of immunogenicity and protective efficacy. This vaccine can be successfully used to provide better protection against circulating IBV in commercial poultry. It may also be a more cost-effective option compared to imported IB vaccines with antigenic heterogeneity.

## List of Abbreviations

IB, infectious bronchitis; IBV, infectious bronchitis virus; ELISA, enzyme linked immune, sorbent assay; SC, subcutaneously; IM, intramuscularly; EID, embryo infective dose; RT-PCR, reverse transcription-polymerase chain reaction.
